# Potential viral pathogenic mechanism in human type 1 diabetes

**DOI:** 10.1007/s00125-014-3340-7

**Published:** 2014-07-30

**Authors:** Darius A. Schneider, Matthias G. von Herrath

**Affiliations:** 1 La Jolla Institute for Allergy and Immunology, 9420 Athena Circle, La Jolla, CA 92037 USA; 2 Department of Medicine, UC San Diego, La Jolla, CA USA; 3 Novo Nordisk Type 1 Diabetes Research Center, Seattle, WA 98109 USA

**Keywords:** Autoimmune, Enterovirus, Environmental factors, Humans, Insulitis, Type 1 diabetes, Viruses

## Abstract

In type 1 diabetes, as a result of as yet unknown triggering events, auto-aggressive CD8^+^ T cells, together with a significant number of other inflammatory cells, including CD8^+^ T lymphocytes with unknown specificity, infiltrate the pancreas, leading to insulitis and destruction of the insulin-producing beta cells. Type 1 diabetes is a multifactorial disease caused by an interactive combination of genetic and environmental factors. Viruses are major environmental candidates with known potential effects on specific key points in the pathogenesis of type 1 diabetes and recent findings seem to confirm this presumption. However, we still lack well-grounded mechanistic explanations for how exactly viruses may influence type 1 diabetes aetiology. In this review we provide a summary of experimentally defined viral mechanisms potentially involved in the ontology of type 1 diabetes and discuss some novel hypotheses of how viruses may affect the initiation and natural history of the disease.

## Introduction

While there is undoubtedly evidence for a genetic basis of type 1 diabetes, especially with regard to permissive HLA class II genotypes, many features of this disease have to be attributed to environmental factors, specifically, (1) the annual increase in type 1 diabetes incidence, currently estimated to be 3% [1]; (2) the strong heterogeneity of its geographical distribution, which is subject to considerable regional gradients [2]; (3) the fact that the incidence rate in first-generation offspring of immigrants is the same as that in the new home country [3, 4].

In animal models of diabetes the established role of innate inflammation in the insulitic process [5–7] and the increasing evidence supporting the contribution of viral infections to a proinflammatory islet milieu [8–10] strongly suggest that viruses may contribute to beta cell damage and dysfunction. The evidence for the presence of similar mechanisms in humans is still circumstantial [11–13]; however, the insights gained from animal studies imply that innate immunity is an important component of the pathogenesis of type 1 diabetes. Lately, novel developments in analysis techniques, as well as access to organ libraries, such as the Network of Pancreatic Organ Donors (nPOD, www.jdrfnpod.org) in the USA or the collection of Foulis et al [14] in the UK will be instrumental in allowing us to link the presence of these environmental determinants to the highly complex histopathological features of type 1 diabetes.

In this review we will analyse how viral infections can account for these highly complex scenarios.

## Hallmarks of type 1 diabetes pathogenesis

**Fig. 1:**
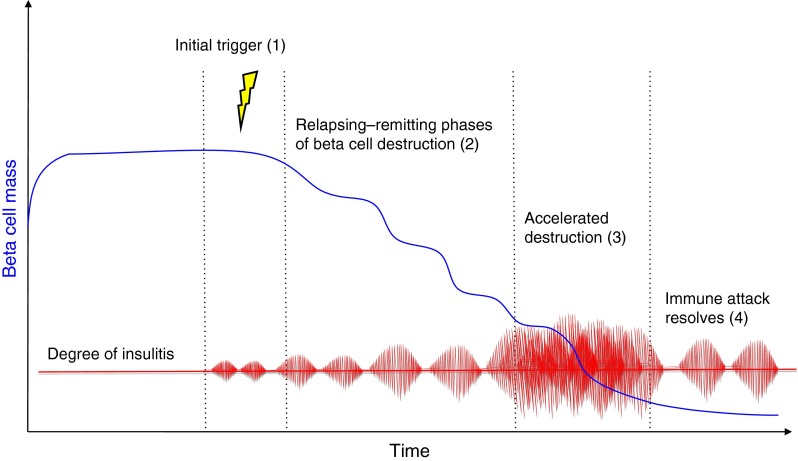
Timeline of development of type 1 diabetes. In genetically susceptible individuals, an as yet unknown environmental trigger (1) causes an underlying inflammation of the pancreas that is characterised by a rather discrete lymphocytic infiltrate and the upregulation of MHC I on some beta cells (insulitis). This represents a fertile soil for a complex interplay between T-effector and T-regulatory cells, eventually favouring the CD8^+^ T-effector-mediated attack, causing a scenario of concomitant beta cell destruction and enhanced proliferation (2). The sequential appearance and spreading of antigenic determinants leads to the enhancement of the immune response (3) and feeds a vicious cycle. As soon as the vast majority of beta cells is destroyed, the immune reaction slows down (4), yet some of its features (MHC I upregulation) remain detectable for a long time, probably fuelled by the few remaining beta cells. Pathophysiologically, phases (2) and (3) coincide with the transition from normoinsulinaemia to hypoinsulinaemia and finally to the loss of detectable C-peptide levels

Type 1 diabetes results from the selective and progressive destruction of insulin-producing cells by autoreactive CD8^+^ T cells [15] and a variable number of bystander CD8^+^ T cells [16, 17]. The presence of anti-islet cell antigen autoantibodies and a predominantly lymphocytic infiltrate of the islets (insulitis) including CD8^+^ T cells antigen-specific for beta cell antigens [15] are regarded as proof of the autoimmune aetiology of the disease [14, 18, 19] (Fig. 1).

### The nature of early disease may be cyclical and relapsing–remitting

Based on the very character of the most prolific feature of type 1 diabetes—insulitis—it is thought that early disease might involve cycling between remission and relapse [20]. In spite of being considered a hallmark of type 1 diabetes, the occurrence of insulitis is heterogeneous and often elusive. When combining all ~215 cases of type 1 diabetes for which minimal clinical and histopathological data exist, it can be estimated that in young type 1 diabetes patients <14 years old, insulitis occurs in 73% of those with a diabetes duration of <1 month, in 60% of those with a diabetes duration of >1 month but <1 year, and in 4% of patients with a diabetes duration of >1 year. In donors >15 years of age, insulitis was only found in 29% of those with acute-onset diabetes [21, 22]. In autoantibody-positive donors, the numbers were far lower: only two out of 62 autoantibody-positive, non-diabetic organ donors showed insulitis [23]. Furthermore, insulitis seems to follow a lobular pattern rather than affecting all islets in a given pancreas [14, 24]. If we regard insulitis as a conditio sine qua non for the later development of type 1 diabetes, given that insulitis is not observed in all cases positive for autoantibodies, we have to consider the possibility that the nature of early disease is remitting–relapsing, with phases of intense inflammation alternating with quiescent phases.

### The distribution of the disease is not homogeneous

Contrary to what we would expect in a stochastic development, the distribution of the immune process in type 1 diabetes is inhomogeneous and lobular, with pancreatic lobules that are affected, and lobules that are not [14, 15, 19, 24, 25]. This pattern is also seen in the map of inflammatory cytokine production [26]. Furthermore, the exocrine pancreas is often smaller [27, 28] and mildly infiltrated, and insulitis is not as prominent as usually seen in animal models, e.g. the NOD mouse [22, 29].

The presence, temporal pattern, cellular composition and distribution of the inflammatory infiltrate [20] are highly variable [30], which is mirrored in the high variability of the preclinical period preceding diabetes [31].

Taken together, we can envision the following picture: an underlying inflammation of the pancreas, caused by an as yet unknown initial event and characterised by a rather discrete lymphocytic infiltrate and the upregulation of MHC I on some beta cells [15], could represent a fertile soil [32, 33] for a complex interplay between effector and regulatory T cells, eventually favouring CD8^+^ effector T cells that attack the beta cells. This leads to a scenario of concomitant beta cell destruction and enhanced proliferation, with the sequential appearance and spreading of antigenic determinants and the enhancement of the immune response [34]. As recent works suggest [35], the underlying inflammatory state of the pancreas might initially involve the exocrine pancreas, prior to the induction of autoimmunity. As soon as the latter is triggered, a vicious cycle is born.

## How can the histopathological hallmarks of diabetes be explained by viral infection?

In spite of a plethora of studies (see Table 1 for an overview of important studies), we are still lacking a clearly established, causal link between viral infection or presence and the development of autoimmunity or progression to diabetes, yet the histopathological hallmarks of diabetes can be elegantly explained as effects of viral interference:

**Table 1:** Synopsis of important studies addressing viral causes of type 1 diabetes

Reference	Virus	Main message
Rasmussen et al, 2011 [90]	Respiratory viruses	Respiratory infections more common in children who later progress to type 1 diabetes.
Beyerlein et al, 2013 [91]	Respiratory viruses, not classified	Increased hazard ratio of islet autoantibody seroconversion is associated with respiratory infections during the first 6 months of life.
Gale, 2008 [92]	Rubella	Congenital rubella may predispose to subsequent autoimmunity but existing studies are weak.
Viskari et al, 2003 [93]	Rubella	No evidence of increased frequency of markers for humoral beta cell autoimmunity in patients with congenital rubella syndrome.
Green et al, 2004 [94]	CVB	Review of 26 case–control studies: no convincing evidence for or against an association between CVB infection and type 1 diabetes.
Stene et al, 2010 [65]	EV	Progression from islet autoimmunity to type 1 diabetes may increase after an EV infection characterised by the presence of viral RNA in blood.
Tapia et al, 2011 [95]	EV	No support for faecal shedding of enteroviral RNA as major predictor of advanced islet autoimmunity.
Yeung et al, 2011 [96]	EV	Clinically significant association between EV infection, detected with molecular methods, and autoimmunity/type 1 diabetes.
Salur et al, 2011 [97]	EV	Nested case–control study where all case children have progressed to type 1 diabetes. EV RNA-positive samples were more frequent among the cases than among the controls.
Oikarinen et al, 2012 [98]	EV	Large proportion of type 1 diabetes patients have prolonged/persistent EV infection associated with an inflammation process in gut mucosa.
Mercalli et al, 2012 [99]	EV	Small intestine biopsy samples from 25 individuals at different stages of type 1 diabetes, 21 controls and 27 individuals with coeliac disease analysed for the presence of EV RNA by in situ hybridisation and RT-PCR. Prolonged/persistent EV infections in gut mucosa are not common in patients with type 1 diabetes.
Viskari et al, 2000 [100]	EV	The rapid decrease in EV infection frequency in Finland may explain the increasing incidence of type 1 diabetes.
Roivainen et al, 2002 [101]	EV	Patterns and consequences of EV infections investigated in cultured adult human isolated islets. The capacity of EV to kill human beta cells or impair their function is not solely defined by the serotype, but also by as yet unidentified characteristics of the virus strain involved.
Viskari et al, 2004 [102]	EV	EV antibodies less frequent in countries with high diabetes incidence compared with countries with low diabetes incidence.
Viskari et al, 2005 [103]	EV	Maternal EV antibodies analysed from serum samples taken from pregnant women between 1983 and 2001 in Finland and Sweden. A low frequency of EV infection in the background population increases the susceptibility of young children to the diabetogenic effect of EV.
Richardson et al, 2009 [104]	EV	EV capsid protein VP1 is commonly found in the islets of recent-onset type 1 diabetic patients, but only rarely in normal paediatric controls.
Gamble et al, 1973 [105]	CVB4	Antibody to CVB4 virus more often found in diabetic patients than in controls, particularly in the 10–19 year age group.
Gamble et al, 1969 [106]	EV	In patients with recent-onset diabetes, no evidence was found of any excess of antibodies to mumps virus or certain common respiratory viruses. However, those diabetics patients who developed insulin dependence within 3 months of onset were found to have higher antibody titres to CVB4.
Dotta et al, 2007 [107]	EV	Pancreatic tissue from six type 1 diabetic and 26 control organ donors analysed via immunohistochemistry, electron microscopy, whole-genome ex vivo nucleotide sequencing, cell culture and immunological studies. CVB4 found in specimens from three of the six diabetic patients.
Laitinen et al, 2013 [62]	CVB1, B3, B6	183 children who persistently tested positive for at least two diabetes-predictive autoantibodies and 366 autoantibody-negative matched control children. CVB1 was associated with an increased risk of beta cell autoimmunity. This risk was strongest when infection occurred a few months before autoantibodies appeared and was attenuated by the presence of maternal antibodies against the virus. Two other coxsackieviruses, B3 and B6, were associated with a reduced risk.
Oikarinen et al, 2014 [61]	CVB1	249 children with newly diagnosed type 1 diabetes and 249 control children matched according to sampling time, sex, age and country recruited in Finland, Sweden, England, France and Greece between 2001 and 2005 (mean age 9 years; 55% male). Antibodies against CVB1 were more frequent among diabetic children than among control children.
Cabrera-Rode et al, 2003 [108]	Echovirus 16	The occurrence of a large-scale echovirus 16 epidemic was associated with the appearance of humoral autoimmune markers of type 1 diabetes. Echovirus 16 infection might be capable of inducing a process of autoimmune beta cell damage.

EV, enterovirus

1. The heterogeneous, possibly relapsing–remitting insulitis, with upregulation of MHC class I molecules on most islets, which persists for years irrespective of the functional status of the islets, can be caused by interferons or other factors secreted from cells infected by virus.
2. Enteroviruses have been shown to have a strong pancreotropism—severe islet damage has been demonstrated in fatal group B coxsackievirus (CVB) infection cases [36], human islets show strong expression of the coxsackie virus and adenovirus receptor (CAR) [37] and beta cells are permissive for enterovirus in vitro [38]. Also, human peripheral blood mononuclear cells (PBMCs) experimentally infected with CVB4 show enhanced production of proinflammatory cytokines such as TNF-α and IL-6 [39].
3. It has recently been shown that the inflammatory state of the pancreas can be explained by direct or indirect viral effects [40, 41].

## Mechanisms of viral involvement in type 1 diabetes aetiology

When we discuss viral infections, we have to consider viral infections of the pancreatic beta cells as well as of those cells adjacent to the beta cells (acinar cells, endothelial cells, neurons) or of cells at a remote location, such as dendritic cells, leading to presentation of cross-reacting epitopes, or of gut cells, leading to increased gut permeability and the presentation of cross-reacting antigens in the pancreatic lymph nodes, where pancreatic and gut lymphatic drainage intersect [42]. From a chronological point of view, viral infections can be either (1) acute, (2) exacerbated chronic infections, (3) reactivated persistent, quiescent viruses (e.g. *Herpesviridae*), or (4) represent the restoration of pathogenicity of viral segments that had long been integrated in the human genome, such as human retroviruses.

### Infection of beta cells

Infection of beta cells with subsequent damage and release of antigens, as well as release of interferons and involvement of both innate and adaptive immune system, is the mechanism by which enteroviruses are thought to be involved in the pathogenesis of type 1 diabetes. This is, by far, the mechanism that has been investigated to the largest extent and has dominated scientific debate on this topic for many years.

#### Acute infection

Acute infection is linked with severe damage of the beta cells and rapid progression towards fulminant diabetes. This is the case in fulminant type 1 diabetes, where an association with direct enteroviral infection has been discussed in case reports [43] and where autoimmunity is not primarily involved. Here we may also add examples of enterovirus-induced diabetes in animals: cattle after infection with the foot and mouth virus [44], voles after infection with *Ljungan virus* [45], rats after infection with the Kilham virus [46], as well as higher non-human primates infected with CVB4 virus [47]. Especially in the case of CVB4, we suspect considerable differences in viral strain-specific pancreotropism.

#### Chronic infection

Chronic, rather slow and persistent type of infection with stimulation of resting beta-cell-antigen-specific T lymphocytes is thought to be the link between CVB infections and the induction of autoimmunity and/or progression to diabetes and leads to subtle changes in the beta cells. These changes may involve the induction of endoplasmic reticulum stress [48] or mutations in tyrosine kinase 2 (TYK2) or similar proteins leading to beta-cell-specific suppression of cytokine responses including interferon [49], which leads to high sensitivity to CVB4 infection [50] or other changes in beta cell metabolism which, in turn, lead to local inflammation and tissue damage with the slow release of sequestered islet antigen and stimulation of quiescent, beta-cell-antigen-specific T (memory) cells.

### Infection of cells adjacent to beta cells

#### Exocrine pancreatic cells

The exocrine pancreas is targeted by a myriad of different viruses, and many of them have been associated with type 1 diabetes either in humans or in animal models: measles virus, congenital rubella virus, mumps virus, cytomegalovirus [51], or even viruses with high penetration of the population, examples of which include influenza A [52] and B [53]. To date, however, we lack a direct causative link between viraemia and the development of autoantibodies or the transition from autoantibody positivity to diabetes, as a recent analysis of cases from The Environmental Determinants of Diabetes in the Young (TEDDY) study has shown [54]. We can envision that a viral infection of acinar cells and the subsequent activation of the innate immune response may be responsible for the inflammatory state that we and others have noticed in the pancreas, which sets the stage for the development of autoimmunity (self meets inflammation). If correct, this might support the application of anti-inflammatory therapies at onset of diabetes (in conjunction with other tolerogenic approaches).

#### Neuronal cells adjacent to beta cells

The pancreatic islet is a highly innervated and vascularised mini organ, and viral infections can occur in any of the cell types surrounding the actual beta cell. The recent observation in the mouse that the first structures to be prone to the autoimmune attack do not necessarily have to be the beta cells themselves, but can be sensory neuronal cells innervating the islet [55], prompted us to investigate the role of neurotropic viruses in the induction of autoimmunity in human samples. *Reoviridae* (like rotavirus) have long been found to induce autoimmune biliary atresia and to infect the plexus myentericus and the nervus vagus and persist in neuroenteric structures, and ciclosporin was shown to inhibit rotavirus replication and to restore interferon beta signalling pathway both in vitro and in vivo [56]. This hypothesis would elegantly account for the lobular spreading and the temporal pattern of diabetes, and also for the methodical difficulties encountered in identifying viral traces in islets, since neurotropic viruses would reside in dorsal root ganglia when quiescent. This concept, corroborated by the fact that remote infections of neurons innervating the myenteric plexus with *human herpesvirus 6* (HHV6) may lead to increased gut inflammation and, subsequently, permeability [57] offers a new avenue for research.

### Infection of cells at remote locations

#### Infection of dendritic cells by viruses carrying cross-reactive epitopes: the concept of molecular mimicry

This mechanism has been extensively discussed elsewhere [58, 59] and is based on the observation that a single T cell receptor can recognise quite distinct but structurally related peptides from multiple pathogens [60]. Directly linked to type 1 diabetes triggering was the observation that one amino acid sequence from GAD65 (PEVKEK) is highly conserved in CVB4 isolates as well as in different viruses of the subgroup of CVB-like enteroviruses.

Coxsackie viruses have long been suspected to be the main culprits in the induction of autoimmunity in the aetiology of type 1 diabetes, and large studies of young children have substantiated these hypotheses: antibodies against CVB1 are more frequent among diabetic children than in control children, while other CVB types do not differ between the groups [61], and CVB1 is associated with an increased risk of beta cell autoimmunity [62]. This risk is strongest when infection occurs a few months before the appearance of autoantibodies and is attenuated by the presence of maternal antibodies against the virus. Two other CVB types, B3 and B6, are associated with a reduced risk, with an interaction pattern suggesting immunological cross-protection against CVB1 [62].

Besides enterovirus, other viruses known to contain cross-reactive epitopes (rubellavirus, rotavirus) have often been identified during the onset of diabetes [63–65] or have been shown to facilitate the appearance of autoantibodies, as in the case of certain strains of echovirus [66].

Pivotal players in these scenarios are the dendritic cells, which are known to initiate the immune response by potent expression of co-stimulatory molecules. The observation that reduced early virus replication blunted CD8^+^ T cell priming and prevented the onset of diabetes in a model of virus-induced diabetes led to the discovery that early virus replication in dendritic cells is essential to disrupt immune tolerance and that this process is dependent on expression of ubiquitin specific peptidase 18 (USP18), an inhibitor of the IFNγ pathway [67]. In this study, early viral replication was only possible in dendritic cells, in which the IFN pathway was downregulated, which is also an important prerequisite for the induction of autoimmunity.

#### Infection of gut cells and changes in gut permeability

This is a speculative thought based on the observation that many viral infections of the gut lead to increased gut permeability, with novel antigens being presented in draining lymph nodes. Bearing in mind that the lower gut and the pancreatic lymphatic drainage intersect in the pancreatic lymph nodes (PLNs) [42], one can easily envision a scenario in which auto-reactive CD8^+^ cells are primed with cross-reactive epitopes occurring in the PLNs as the result of increased gut permeability. Another possibility is that changes in the microbiome are induced by viral infections. For example, it is known that, in particular, bacteriophage, i.e. viruses that infect bacteria of the human gut microbiome, show high variation as a result of changes in diet, hormonal balance or even climate changes [68]—much more than the infected bacteria themselves. Recent findings suggest that altering certain bacterial populations present in the gut can lead to an inflammatory state associated with Th1/Th17 polarisation and, thus, to autoimmunity [69]. Therefore, since bacteriophage seem to be important determinants of the gut microbiome, the differences in diabetes incidence between monozygotic twins, or between inhabitants, different regions [2], or between immigrants and the first-generation of offsprings from those immigrants [3, 4] can be explained by differences in the gut virome.

## Other mechanisms potentially involved in virally facilitated initiation or acceleration of autoimmunity

### Reactivation of endogenous HERV

Human endogenous retroviruses (HERVs) [70] are fossil viruses that began to be integrated into the human genome some 30–40 million years ago and now make up 8% of the genome. HERVs may be triggers of autoimmune disease by provision of a source of novel viral genes for recombination with exogenous viruses, by immune dysregulation or by super-antigen motifs. HERV infection has been shown to trigger autoimmune rheumatic disease, and the resultant inflammation observed could lead to elevated HERV expression [70]. The potential role of HERV in diabetes has not been deciphered yet, but we will likely see more work done in this field in the upcoming years.

### Activation of polyclonal T cells

Recent observations in a virus-based diabetes models of the mouse [17, 71, 72] have taught us that beta-cell-antigen-specific T cells can recruit a high number of non-beta-cell-antigen-specific bystander T cells that add to the destruction of beta cells (more than 98% of infiltrating CD8 cells in the rat insulin promoter–lymphocytic choriomeningitis virus (RIP-LCMV) model are not viral-antigen-specific [17]). From this point of view, any viral infection of pancreatic structures can lead to an accumulation of activated T cells in the immediate vicinity of beta cells, which might significantly affect their health and function.

### Viral transformation of autoreactive B cells

Polyreactivity can arise as a result of random rearrangement of Ig genes during B cell development [73], yet most of the autoreactive B cells are eliminated via clonal deletion, anergy or receptor editing [74]. However, cross-reactivity is a common serological feature of certain viral infections in humans (HIV [75], Epstein–Barr virus [76], hepatitis A virus [77], hepatitis C virus [78]) and persisting viral infection have long been shown to lead to polyclonal B cell activation [79]. We have yet to find an explanation of how these phenomena are accompanied by a mechanism for affinity maturation of these clonal products, which would be the crucial prerequisite for autoimmunity, but recent observations have provided new, exciting insights [80, 81].

## Can viral infections afford protection against diabetes?

In the NOD mouse, we and others have shown that infection with CVB can abrogate the development of type 1 diabetes [82] when given at a very early time point. Mechanistic explanations comprise an upregulation of programmed death ligand 1 (PD-L1) and TNF-α production, as well as a bystander activation of protective regulatory T cells. Furthermore, transferring a small number of regulatory T cells (that would not normally be sufficient to afford protection) from a NOD mouse that has previously been infected with CVB3 to another NOD mouse will protect the latter from developing type 1 diabetes; thus, the viral infection may invigorate the regulatory T cell compartment [82]. These enhancing effects upon polyclonal Tregs are mainly elicited through TLR2 [83].

Conclusive information in humans is lacking. A prerequisite for viral infection-associated induction of autoimmunity is the ability of the viral strain to damage islet cells and to induce proinflammatory innate immune responses within the infected islets. Thus, the presence of certain viral strains that lack this ability (as is the case with CVB6 or echovirus E4 [66]) may protect the host from infections with their beta cytotoxic counterparts. Hence, there might be a protective mechanism at play, similar to the one facilitated by the commensal bacteria present in the gut, and further work is necessary to ascertain this.

Besides strain specificities, infection dose and viral replication rate may also determine whether a given infection is protective or promoting with regard to the initiation of autoimmunity [84]. These differential effects may be explained by differences in the virus-mediated upregulation of inflammatory cytokine production, since the potentiation of cytokine production in infected human PBMCs has been shown to be associated to CVB4 infectivity. Hence, a vaccine covering all major CVB strains, for example, might lower the severity of infections and transform a promoting into a protective infection type.

## Conclusions

When cumulative environmental determinants facilitate the development of autoimmunity, virus infections may serve as one of many risk factors. While many associations have been found between type 1 diabetes and viruses, mostly enteroviruses, we do not have a clear picture overall. It remains to be determined how often viruses induce autoimmunity or beta cell destruction and how often they accelerate the progression from autoimmunity to disease.

Attempts to directly demonstrate the presence of viral peptides within beta cells of diabetic patients have led to controversial results. In spite of several successful reports [85], a recently published work by Korsgren and colleagues [86] has reported that the particular mouse monoclonal antibody (clone 5D8/1) used to detect the viral capsid protein VP1 strongly cross-reacts with human mitochondrial peptides, especially in situations of mitochondrial stress. Thus, in addition to the detection of viruses by antibodies, we will need to demonstrate the presence of their genome by in situ hybridisation and, in the ideal case, associate these findings with local pathology present in the human pancreas, such as the lobular MHC class I upregulation in whole islets—indeed, such effort is already being carried out by the nPOD viral consortium (nPOD-V) [87] and should provide more conclusive results within the next year. The recently founded nPOD-V group represents an unparalleled collaborative setting, highly committed to answer the question of viral implication in diabetes and we are looking forward to the results.

From a mechanistic point of view, viral infections can very well explain many of the hallmark features of early diabetes, the difficult task is now to focus on novel methodologies that are capable of dissecting the few existing specimens in depth and establish conclusive associations between pathology and viral presence or traces thereof. The breakdown of tolerance towards autoantigens could indeed represent a derailment of physiological autoreactivity and may thus be a secondary phenomenon caused by chronic stimuli. One of these stimuli might be chronic viral infections, but we have to bear in mind that there are other chronic states, such as the recently described regurgitation of duodenal bacteria into the common pancreatic duct [88] or various forms of stress [89], that can serve as stimuli in this very context.

For the future, we need considerably more studies on human specimens to address crucial questions in the quest for the causes of type 1 diabetes: What is the exact phenotype and antigen specificity of immune cells infiltrating the islets? What is the exact involvement of the innate immune systems in human type 1 diabetes? What is the exact time point for the occurrence of insulitis? What methods are most suitable to detect viral causes of autoimmunity and how can we avoid causality traps in complex biological systems? What changes within the islets may lead to their own demise even before autoimmunity is involved?

## Abbreviations

CVB: Group B coxsackievirus
HERV: Human endogenous retrovirus
nPOD: Network of Pancreatic Organ Donors
PBMC: Peripheral blood mononuclear cell
PLN: Pancreatic lymph node
